# Hybrid Management of Dysphagia Lusoria with Tevar Implantation and Bilateral Subclavian Arteries Debranching: A Review of the Literature and a Case Report

**DOI:** 10.3390/jpm14060547

**Published:** 2024-05-21

**Authors:** Ovidiu Stiru, Mircea Robu, Pavel Platon, Serban-Ion Bubenek-Turconi, Vlad Anton Iliescu, Catalina Parasca

**Affiliations:** 1 Faculty of Medicine, Carol Davila University of Medicine and Pharmacy, 050474 Bucharest, Romania; ovidiu.stiru@umfcd.ro (O.S.); bubenek@alsys.ro (S.-I.B.-T.); vladanton.iliescu@gmail.com (V.A.I.); catalina.parasca@gmail.com (C.P.); 2 Department of Cardiac Surgery, “Prof. Dr. C. C. Iliescu” Emergency Institute for Cardiovascular Diseases, 022322 Bucharest, Romania; 3 Catheterization Laboratory, “Prof. Dr. C. C. Iliescu” Emergency Institute for Cardiovascular Diseases, 022328 Bucharest, Romania; pavelplaton@yahoo.com; 4 1st Department of Cardiovascular Anesthesiology and Intensive Care, “Prof. Dr. C. C. Iliescu” Emergency Institute for Cardiovascular Diseases, 022328 Bucharest, Romania

**Keywords:** dysphagia lusoria, aberrant subclavian artery, endovascular repair, carotid–subclavian artery bypass

## Abstract

Aberrant right subclavian artery (ARSA) causing dysphagia, the so-called “dysphagia lusoria”, is a frequent embryologic anomaly of the aortic arch. In symptomatic patients, studies report several management options including surgical, hybrid, and totally endovascular strategies. Hybrid techniques have the advantage of no chest opening with reduced morbidity, but the problem of the ARSA stump causing recurrent or persistent dysphagia remains challenging in some cases. We conducted a literature review on the management strategies of ARSA and presented the case of a 72-year-old female patient with ARSA and dysphagia managed with thoracic endovascular repair of the aorta (TEVAR) and bilateral carotid–subclavian artery bypass. This technique was chosen because of the severe calcifications at the level of ARSA origin that would make surgical ligation difficult, or if an occluder device not suitable. We think that a patient-tailored approach should be considered in cases of dysphagia lusoria, considering that a multitude of strategies are reported.

## 1. Introduction

“Dysphagia lusoria”, also known as Bayford syndrome, was first introduced by David Bayford in 1794 and describes an extrinsic compression of the esophagus caused by a vascular anomaly of the aortic arch [1]. The most frequent embryologic abnormality of the aortic arch is an aberrant right subclavian artery (ARSA) [2]. When ARSA is present, the innominate artery is absent and four arteries arise from the aortic arch: the right common carotid artery, the left common carotid artery, the left subclavian artery, and finally the right subclavian artery, called the “arteria lusoria”. Although the typical course is retroesophageal in its course to the right arm, pretracheal trajectory is also described [3]. In 20 to 60% of cases, ARSA is associated with an aneurismal dilatation at the origin from the aortic arch, known as Kommerell’s diverticulum (KD) [4].

Most cases of ARSA are asymptomatic and are discovered in imaging studies. Symptoms are reported in 7–10% of adults with this anomaly [5]. In adults, compression of the esophagus can lead to dysphagia, especially when a common carotid trunk is present [6].

Because there are no definitive guidelines for the treatment of “dysphagia lusoria”, there is a high degree of heterogeneity between the preferred treatments. These methods include a population of patients with chest opening using open or hybrid surgery through sternotomy/thoracotomy and another population of patients wherein chest or mediastinum opening was avoided with endovascular or hybrid treatment [7]. All these techniques are reported to have low mortality rates [7]. When considering the hybrid approach, closing the origin of ARSA is a key part because a perfused subclavian stump can cause persistent or recurrent dysphagia [8]. Several options are reported, such as occluder devices, and coil embolization of a endovascular repair of the aorta with an endostent without any clear indications.

We describe a case of “dysphagia lusoria” in a 72-year-old female patient with severe calcifications at the origin from the aorta, managed successfully with hybrid therapy consisting of TEVAR and bilateral carotid–subclavian artery bypass in the same setting.

## 2. Case Report

**Figure 1:**
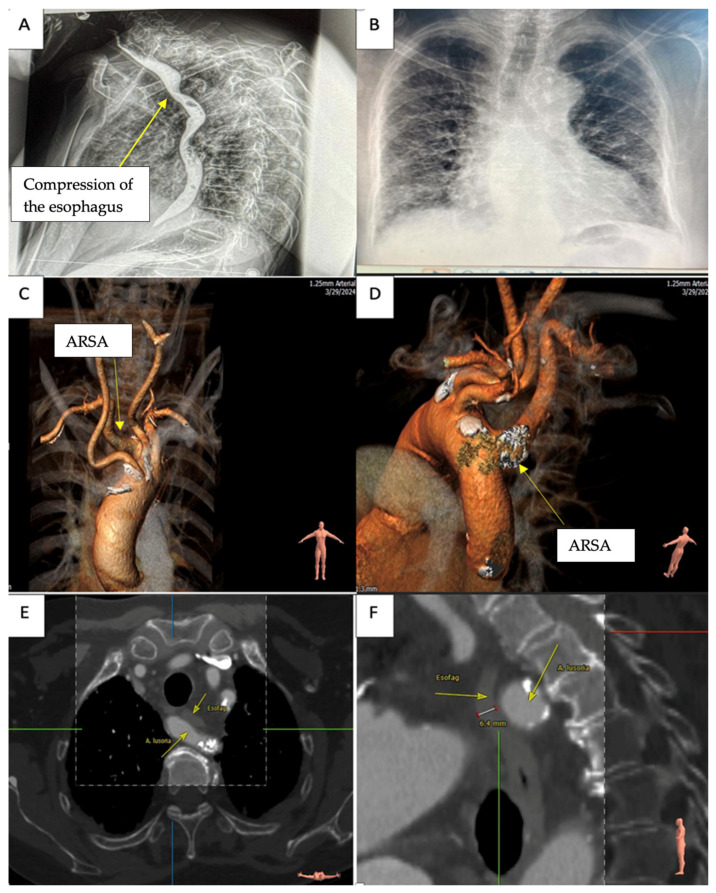
(**A**) Barium swallow study with esophageal compression at the level of the left clavicle. (**B**) Chest XR showing cardiomegaly and diffuse bilateral reticular and micronodular interstitial syndrome. (**C**,**D**) ARSA with calcifications at the origin (yellow arrows). (**E**,**F**) Cross- and sagittal sections showing compression of the esophagus by ARSA (yellow arrows); esofag—esophagus; A lusoria—aberrant right subclavian artery.

A 72-year-old female patient with a history of prolonged progressive dysphagia in the last 5 years and 20 kg weight loss in the last year was admitted to our center. The patient was diagnosed with dysphagia lusoria in a tertiary center, where a chest CT angiography revealed an ARSA. The patient could only swallow liquids and was in poor physical condition with virtually no effort tolerance and intense fatigue. She was 40 kg and 165 cm in height. Past medical history revealed arterial hypertension and smoking. Clinical examination was within normal limits, the patient was stable, and her systolic blood pressure was 100–110 mmHg with a 110 bmp regular pulse and a 98% peripheral oxygen saturation. Blood tests revealed a mild anemic syndrome. The electrocardiogram showed normal sinus rhythm without any ST-T changes. Chest XR (Figure 1B) revealed cardiomegaly as well as diffuse bilateral reticular and micronodular interstitial syndrome without pleural effusion. We interpreted the pulmonary modifications secondary to previous COVID-19 respiratory infection. Transthoracic echocardiography showed normal biventricular function, no significant valvular pathology, and no pericardial effusion. Another cervical and chest CT angiography was obtained, confirming the presence of an aberrant subclavian artery, in our case a right subclavian artery with a retroesophageal course (Figure 1C). The ascending aorta, aortic arch, and descending thoracic aorta had a normal caliber. The ARSA presented a moderate dilatation at the origin with intense calcifications extended 3 cm from the beginning of the artery (Figure 1D). Cross- and sagittal sections (Figure 1E,F) on chest CT show compression of the esophagus. The residual lumen of the esophagus was 6.4 mm in diameter where the ARSA comes into contact. To better evaluate the esophageal stenosis, a barium swallow study was performed (Figure 1A). The results clearly show a compression of the esophagus just beneath the level of the left clavicle.

Considering the long history of dysphagia and weight loss, the decision was made to undertake hybrid management of the patient.

### Surgical Technique

**Figure 2:**
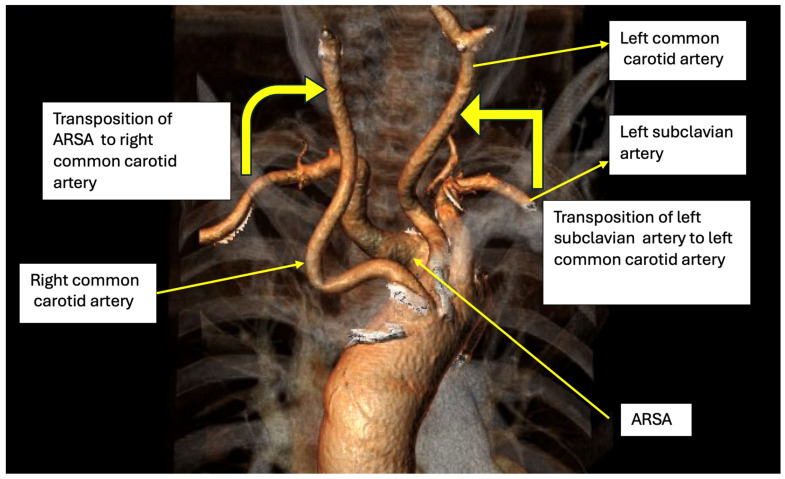
Operating plan.

Considering the considerable risk associated with an exclusively surgical approach for the treatment of severely symptomatic dysphagia lusoria in this frail patient, a multidisciplinary decision was made in favor of a hybrid approach. After careful analysis of the specific anatomy, a treatment plan was proposed: endovascular exclusion of the aberrant right subclavian artery and bilateral carotid to subclavian bypass (Figure 2). The patient’s informed consent was obtained, and she was prepared for surgery.

The patient was placed under general anesthesia with continuous invasive hemodynamic monitoring and continuous non-invasive measurement of cerebral oxygen saturation. The monitoring of cerebrospinal fluid pressure was not necessary.

First, a 22F delivery system was introduced via the right femoral artery. A Valliant thoracic endoprosthesis (Medtronic AVE, Santa Rosa, CA, USA) measuring 100 mm in length with a proximal diameter of 32 mm and a distal diameter of 32 mm was deployed into the aortic arch to occlude the origin of the ARSA. As anticipated from the preoperative CT planning the left subclavian artery was also covered by the endoprosthesis because the measured seal zone from the left subclavian artery was only 14 mm and increased to 29 mm when measured from the left carotid artery.

**Figure 3:**
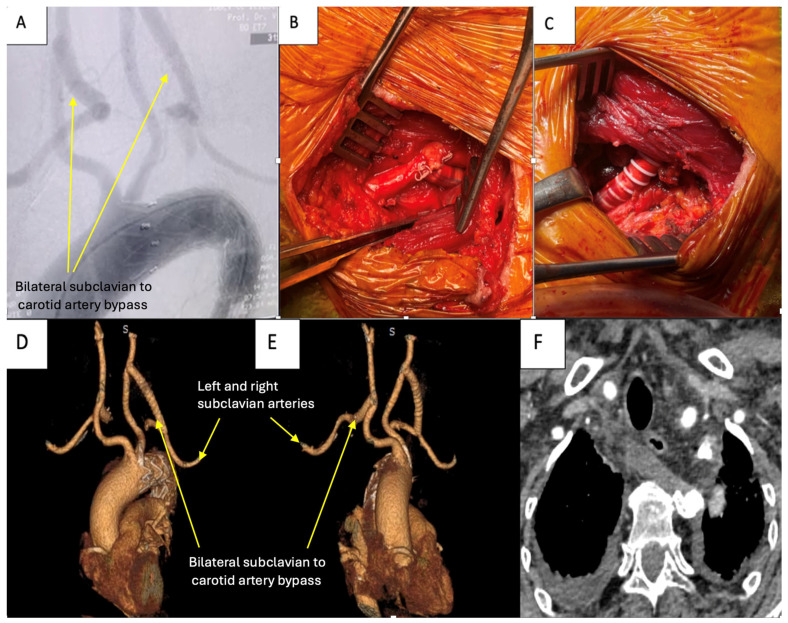
(**A**) Arteriography showing bilateral subclavian carotid bypass. (**B**) Intraoperative view of right carotid–subclavian bypass. (**C**) Intraoperative view of left carotid–subclavian bypass. (**D**) Postoperative chest CT angiography reconstruction of the aorta and supra-aortic vessels. (**E**) A 1-month chest CT angiography reconstruction of the aorta and supra-aortic vessels. (**F**) Postoperative chest CT cross-section showing no compression on the esophagus.

In the next step, the right common carotid artery (CCA) and the right subclavian artery (SA) were exposed through a low longitudinal cervical incision, extending laterally in the supraclavicular fossa. A tunnel beneath the sternocleidomastoid muscle was created from the carotid to the axillary artery. Intravenous heparin was administered for an activated clotting time of 250 s. An 8 mm ringed ePTFE graft (W. L. Gore, Flagstaff, Ariz) was anastomosed to the lateral aspect of the CCA, followed by an end-to-end anastomosis with the distal right SA (Figure 3B). The proximal end of the right SA was sutured below the level of the clavicle. In a similar fashion, left CCA and left SA were exposed through a low cervical incision. A carotid to subclavian bypass was performed using another 8 mm ringed ePTFE graft (W. L. Gore, Flagstaff, Ariz) tunneled under the sternocleidomastoid muscle by using end-to-side anastomoses on both the left CCA and left SA (Figure 3C). The proximal left SA was tied off.

Arteriography at the end of the procedure showed permeable carotid arteries, exclusion of both subclavian arteries in the proximal segments, and patent bilateral carotid to subclavian bypasses.

The total procedure time was approximately 175 min, with a fluoroscopy time of 20 min (Figure 3A,D).

The patient was extubated 4 h after intensive care arrival and transferred to the surgical ward the next day. She confirmed the resolution of dysphagia. No esophageal compression was observed on the chest CT performed postoperatively (Figure 3F). No neurological dysfunction was recorded postoperatively. After transfer to the ward, the patient had an uneventful recovery and was discharged 5 days post-procedure. At 1-month follow-up, a cervico-thoracic CT angiography was performed, which showed exclusion of both subclavian arteries in the proximal segments and patent bilateral carotid to subclavian bypasses (Figure 3E). At 3-month follow-up visit, the patient had no dysphagia complaints and had gained 5 kg.

## 3. Discussion

We presented a case of ARSA with a long history of dysphagia and weight loss, managed successfully with TEVAR and bilateral subclavicular carotid bypass. While this particular strategy that combined endovascular and surgical techniques is well described for the management of ARSA, there are limited reports of bilateral subclavian carotid bypass. We found only one study that reported four cases of bilateral carotid–subclavian artery bypass combined with TEVAR [9]. In two of the patients, dysphagia was not resolved due to persistent compression. They concluded that ascending aorta–subclavian artery bypass and thoracic open aortic repair are the best management for these patients. Another study reports that bilateral revascularization is more frequent in patients with an endovascular management of ARSA [10].

For our patient in particular, the close proximity of the ARSA and the left subclavian artery mandated that a left subclavian carotid bypass should also be performed. The measured seal zone from the left subclavian artery in our case was only 14 mm and increased to 29 mm when measured from the left carotid artery. The rationale for choosing TEVAR was the following: first, a hybrid approach excludes the need for open chest surgery with lower morbidity; second, we considered the risk of wall erosion if we had used an occluder device because of calcification at the origin of ARSA [11]; third, we considered that surgical ligation of the origin of the ARSA was not a solution considering the severe calcifications present at this level.

In this case, the choice of TEVAR and bilateral subclavicular carotid bypass proved to be a successful option for the patient with the resolution of dysphagia in the postoperative period and at serial follow-ups. Also, chest CT angiography confirmed the absence of esophageal compression. Complications reported in the literature, such as pleural effusion, plexus brachialis injury, or recurrent laryngeal nerve paralysis, did not occur.

Another aspect is timing of the intervention, especially in old patients. While there are no guidelines, most authors consider symptomatic ARSA to require treatment. Our patient was 72-years-old, and one should question if ARSA should not have been managed early in her life with lower surgical risks. Indeed, severe symptoms occurred in the last 5 years, but we think that operating at an early stage when symptoms are mild/moderate reduces the risk of such interventions.

### Review of Literature

Congenital abnormalities of the aortic arch are described in 25% of imaging studies [7]. The bovine arch is the most frequent congenital abnormality, while an aberrant subclavian artery (ASA) has an estimated prevalence of 0.8–1% [12]. In a normal left artery, ASA affects the right subclavian artery, which arises as a fourth vessel and usually has a retro-esophageal course [3]. ASA can be associated with an aneurismal degeneration at the vessel origin, described as Kommerell’s diverticulum (KD) [13].

There is a difference in symptomatology in the pediatric population versus the adult population. Respiratory symptoms like wheezing, stridor recurrent pneumonia, and cyanosis are reported in infants because the trachea is compressible. In adults, respiratory symptoms are rare and the classical finding is dysphagia because the trachea is more rigid [6]. Other symptoms include hoarseness, dyspnea, and chest or back pain. High incidences of dissection or rupture are reported even in asymptomatic patients [14]. Other possible mechanisms for “dysphagia lusoria” besides extrinsic compression are aneurysm formation, elongation of the aorta, increased rigidity of the artery from atherosclerosis, and the coexistence of ARSA with truncus bicaroticus [15].

There are no formal guidelines for the management of ARSA, so it is to be expected that a variety of proposed treatments are reported. Most authors agree that symptomatic patients need surgery, while in asymptomatic patients, a KD of more than 3 cm or associated aortic pathology should warrant surgery [7].

In a systematic review of 732 patients, the three most common solutions reported in 555 patients were open surgical aortic treatment with cardiopulmonary bypass; cervical debranching with thoracic endovascular aortic repair (TEVAR); and supraclavicular cervical debranching and ASA ligation or plug occlusion [7]. In the group treated with open or hybrid procedures using sternotomy/thoracotomy, the mortality rate was 1.62% and symptom relief was 99.52%. In the endovascular or hybrid treatment without chest opening group, mortality was 1.96% and symptom relief was 95.79%. However, the strategy consisting of supraclavicular debranching and ASA ligation or plug occlusion was the strategy associated with virtually no complications when considering spinal stroke ischemia, arm ischemia, major stroke, or death.

Traditionally, open surgery has been the treatment of choice. The first surgical technique reported was performed by Gross in 1946. He successfully ligated an ARSA in a 4-month-old baby via a left thoracotomy [16]. However, the risk in arm ischemia or subclavian steal phenomenon mandated some kind of flow restoration technique. This was first performed by Bailey in 1965, who performed a reimplantation of ARSA into the aortic arch in an adult [16]. Restoring pulsatile flow to the right subclavian artery is currently realized by anastomosis of the divided subclavian artery to the ascending aorta or the right common carotid artery, either directly or by the use of a graft [17]. Currently, there is no standard approach. Left and right thoracotomy, median sternotomy, cervical incisions, or a combination of these are reported. In a systematic review including 33 cases, Kiefffer reported that the surgical approach should be tailored according to the presence or absence of KD and or aneurismal dilatation of the aortic arch/descending aorta, proposing an ASA classification [14]. The first group included patients with dysphagia caused by ARSA without KD. Surgical procedures in this category consisted of isolated cervicotomy, or a cervicotomy associated with contralateral posterolateral thoracotomy. Thoracotomy was necessary either for the treatment of concomitant atherosclerotic lesions of the arch vessels or because dissection of the esophagus or ARSA was not achievable only through cervicotomy alone, usually in the case of patients with narrow thoracic inlet. In this group, flow to the right subclavian artery was in most cases achieved with direct reimplantation of the ligated ARSA into the right common carotid artery or ascending aorta. Another group is composed of patients with symptomatic occlusive disease of ARSA without a KD. Only a cervical approach was necessary in this group. Reimplantation directly into the common carotid artery was used in all cases. Group three is represented by ARSA with KD without aortic arch or descending aorta lesions, while the last group represents ARSA with aneurismal dilatation of the aortic arch/descending aorta with or without KD. For ARSA with KD, the authors report only a cervical approach, with reimplantation of ARSA into the right common carotid artery. For associated aortic arch aneurism, cervical incision associated with sternotomy and or thoracotomy is described.

For adult patients, the literature suggests that in RASA without KD, the supraclavicular approach is ideal for exposure and treatment [18,19,20]. Thoracotomy may be more suitable for ARSA without KD originating from the post-eromedial side of the aortic arch/distal aorta when considering mobilization and reimplantation. A right thoracotomy may also reduce the risk of laryngeal nerve damage, especially damage to the left recurrent nerve, which is close to the origin of ARSA [20].

Endovascular advances offer hybrid treatments of dysphagia lusoria. These endovascular techniques (TEVAR, embolization of the origin of ARSA, vascular plug) necessitate a subclavian to carotid transposition or carotid–subclavian bypass with Dacron via a supraclavicular incision. The main advantage of these techniques is that there is no need for chest opening, reducing the morbidity associated with this procedure [8]. Another advantage is the exclusion of the ARSA stump by the endovascular approach. In time, it can become aneurismal and cause persistent compression on the esophagus or the distal aortic arch, with recurring symptoms [8].

Several reports describe a hybrid procedure that involves a right subclavian artery to right carotid artery transposition through a right supraclavicular incision in the first stage and exclusion of the origin of the ARSA with a vascular plug (Amplazer device) via the right femoral artery in the second stage. The only reported postoperative complication is phrenic nerve injury with elevation of the right hemidiaphragm with rapid improvement afterward [21,22]. Another option for the exclusion of the ARSA stump after subclavian transposition or bypass is embolization of the origin of ARSA using coils [23]. Closing the origin of ARSA with TEVAR is another option, and several authors have reported this strategy [24,25,26]. The reports raise a concern in Marfan patients when using TEVAR because of the high rate of reinterventions [11]. However, TEVAR could be safer when considering the risk of erosive complications associated with septal Amplazer occluders, even years after their placement. Multiple reports describe cases where these rigid devices erode the vessel wall and cause esophageal fistulas [11]. Also, there is a risk of device migration into the distal aorta, and recommendations for 50% oversizing exist, despite the greater risk of wall erosion [27].

A fully percutaneous approach is also reported without the need for surgery. Based on computed tomography images, Gafoor et al. implanted a custom-made endovascular graft. This prosthesis was inserted distal to the origin of the right common carotid artery, had fenestrations to preserve the origins of the left common carotid artery and left subclavian artery, and had a separate fenestration that allowed for insertion of a stented endograft into the ARSA [28]. Another strategy reported for dysphagia lusoria without surgical intervention is endoscopic dilatation of the esophageal stricture. However, the author states that this technique only temporarily relieves the dysphagia [28]. In cases of persistent dysphagia, La Regina reports robotic-assisted resection of the right subclavian stump [29].

In the case of ARSA associated with KD, Bloom et al. propose a treatment algorithm based on clinical presentation and patient factors. They propose that symptomatic patients should undergo surgical management, as with patients with aortic emergency or those with inadequate landing zones. Symptomatic patients with adequate landing zones should undergo endovascular repair [30]. For patients with surgical management, left heart bypass or hypothermic circulatory arrest are reported. However, late outcomes were not affected by the choice of treatment [10].

## 4. Conclusions

We presented the case of a 72-year-old patient with ARSA and dysphagia lusoria successfully managed with a hybrid approach. While various surgical, hybrid, and totally endovascular treatment strategies are reported, we think that a patient-tailored strategy should be planned. In our case, severe calcifications at the origin of the ARSA pointed toward hybrid management with endovascular aortic repair. We considered the risk of wall erosion if we had used an occluder device, and surgically suturing the origin of ARSA would have also been challenging. The measured seal zone from the left subclavian artery in our case was only 14 mm and increased to 29 mm when measured from the left carotid artery, so a bilateral carotid–subclavicular bypass was performed after TEVAR implantation. The patient evolution was without any periprocedural complication, and she necessitated only 24 h of intensive care surveillance and was discharged on postoperative day 5. She confirmed resolution of the dysphagia and serial follow-ups were favorable. Considering the favorable evolution of this case, we consider this a safe strategy for patients with ARSA and severe calcified origin.

## Data Availability

Data available on request.

## References

[1] Nedelcu A.H., Lupu A., Moraru M.C., Tarniceriu C.C., Stan C.I., Partene Vicoleanu S.A., Haliciu A.M., Statescu G., Ursaru M., Danielescu C. (2024). Morphological Aspects of the Aberrant Right Subclavian Artery—A Systematic Review of the Literature. J. Pers. Med..

[2] Molz G., Burri B. (1978). Aberrant subclavian artery (arteria lusoria): Sex differences in the prevalence of various forms of the malformation. Evaluation of 1378 observations. Virchows Arch. A Pathol. Anat. Histol..

[3] Chen J., Liu L., Kou X., Wang C. (2023). Case report: Right vertebral and carotid artery anomalies with an aberrant right subclavian artery in two patients. Front. Neurol..

[4] van Rosendael P.J., Stöger J.L., Kiès P., Vliegen H.W., Hazekamp M.G., Koolbergen D.R., Lamb H.J., Jongbloed M.R.M., Egorova A.D. (2021). The Clinical Spectrum of Kommerell’s Diverticulum in Adults with a Right-Sided Aortic Arch: A Case Series and Literature Overview. J. Cardiovasc. Dev. Dis..

[5] Delap T.G., Jones S.E., Johnson D.R. (2000). Aneurysm of an aberrant right subclavian artery presenting as dysphagia lusoria. Ann. Otol. Rhinol. Laryngol..

[6] Polguj M., Chrzanowski Ł., Kasprzak J.D., Stefańczyk L., Topol M., Majos A. (2014). The aberrant right subclavian artery (arteria lusoria): The morphological and clinical aspects of one of the most important variations—A systematic study of 141 reports. Sci. World J..

[7] Konstantinou N., Antonopoulos C.N., Tzanis K., Kölbel T., Peterß S., Pichlmaier M., Stana J., Tsilimparis N. (2022). Systematic Review and Meta-Analysis of Outcomes after Operative Treatment of Aberrant Subclavian Artery Pathologies and Suggested Reporting Items. Eur. J. Vasc. Endovasc. Surg..

[8] Jalaie H., Grommes J., Sailer A., Greiner A., Binnebösel M., Kalder J., Schurink G.W., Jacobs M.J. (2014). Treatment of symptomatic aberrant subclavian arteries. Eur. J. Vasc. Endovasc. Surg..

[9] Selçuk İ., Sicim H., Selçuk Ü.N., Güven B.B., Yılmaz A.T. (2022). Three Different Strategies for Repair of Symptomatic or Aneurysmatic Aberrant Right Subclavian Arteries. Braz. J. Cardiovasc. Surg..

[10] Bloom J.P., Attia R.Q., Sundt T.M., Cameron D.E., Hedgire S.S., Bhatt A.B., Isselbacher E.M., Srivastava S.D., Kwolek C.J., Eagleton M.J. (2021). Outcomes of open and endovascular repair of Kommerell diverticulum. Eur. J. Cardiothorac. Surg..

[11] Crawford G.B., Brindis R.G., Krucoff M.W., Mansalis B.P., Carroll J.D. (2012). Percutaneous atrial septal occluder devices and cardiac erosion: A review of the literature. Catheter. Cardiovasc. Interv..

[12] Müller M., Schmitz B.L., Pauls S., Schick M., Röhrer S., Kapapa T., Schlötzer W. (2011). Variations of the aortic arch—A study on the most common branching patterns. Acta Radiol..

[13] van Son J.A., Konstantinov I.E., Burckhard F. (2002). Kommerell and Kommerell’s diverticulum. Tex. Heart Inst. J..

[14] Kieffer E., Bahnini A., Koskas F. (1994). Aberrant subclavian artery: Surgical treatment in thirty-three adult patients. J. Vasc. Surg..

[15] Ulger Z., Ozyürek A.R., Levent E., Gürses D., Parlar A. (2004). Arteria lusoria as a cause of dysphagia. Acta Cardiol..

[16] Gross R.E. (1946). Surgical Treatment for Dysphagia Lusoria. Ann. Surg..

[17] Atay Y., Engin C., Posacioglu H., Ozyurek R., Ozcan C., Yagdi T., Ayik F., Alayunt E.A. (2006). Surgical approaches to the aberrant right subclavian artery. Tex. Heart Inst. J..

[18] Orvald T.O., Scheerer R., Jude J.R. (1972). A single cervical approach to aberrant right subclavian artery. Surgery.

[19] Valentine R.J., Carter D.J., Clagett G.P. (1987). A modified extrathoracic approach to the treatment of dysphagia lusoria. J. Vasc. Surg..

[20] Taylor M., Harris K.A., Casson A.G., DeRose G., Jamieson W.G. (1996). Dysphagia lusoria: Extrathoracic surgical management. Can. J. Surg..

[21] Cobos-González E., Aragón-López J.A., García Buen-Abad R., Rojas J.A., Gutiérrez A. (2016). Hybrid treatment of dysphagia lusoria: Right carotid to subclavian bypass and endovascular insertion of an Amplatzer II Vascular Plug. Rev. Esp. Enferm. Dig..

[22] Leon M., Garibaldi M., Virgen F., Ramírez-Cerda C., Cohen-Mussali S. (2020). Hybrid Treatment of Aberrant Right Subclavian Artery Causing Dysphagia Lusoria by Subclavian to Carotid Transposition and Endovascular Plug. Vasc. Spec. Int..

[23] Sigdel A., Wayne E.J., Dwivedi A.J. (2020). Hybrid Endovascular Treatment of Dysphagia Lusoria: Report of 2 Cases. Vasc. Endovascular Surg..

[24] Kopp R., Wizgall I., Kreuzer E., Meimarakis G., Weidenhagen R., Kühnl A., Conrad C., Jauch K.W., Lauterjung L. (2007). Surgical and endovascular treatment of symptomatic aberrant right subclavian artery (arteria lusoria). Vascular.

[25] van Bogerijen G.H., Patel H.J., Eliason J.L., Criado E., Williams D.M., Knepper J., Yang B., Deeb G.M. (2015). Evolution in the Management of Aberrant Subclavian Arteries and Related Kommerell Diverticulum. Ann. Thorac. Surg..

[26] Davidian M., Kee S.T., Kato N., Semba C.P., Razavi M.K., Mitchell R.S., Dake M.D. (1998). Aneurysm of an aberrant right subclavian artery: Treatment with PTFE covered stentgraft. J. Vasc. Surg..

[27] Morris M.E., Benjamin M., Gardner G.P., Nichols W.K., Faizer R. (2010). The use of the Amplatzer plug to treat dysphagia lusoria caused by an aberrant right subclavian artery. Ann. Vasc. Surg..

[28] Gafoor S., Stelter W., Bertog S., Sievert H. (2013). Fully percutaneous treatment of an aberrant right subclavian artery and thoracic aortic aneurysm. Vasc. Med..

[29] La Regina D., Prouse G., Mongelli F., Pini R. (2020). Two-step treatment of dysphagia lusoria: Robotic-assisted resection of aberrant right subclavian artery following aortic debranching. Eur. J. Cardiothorac. Surg..

[30] Bogliolo G., Ferrara M., Masoni L., Pietropaolo V., Pizzicannella G., Miscusi G. (1987). Dysphagia lusoria: Proposal of a new treatment. Surg. Endosc..

